# “Five-year changes in population newborn health associated with new preventive services in targeted risk-group pregnancies”

**DOI:** 10.1186/s12913-019-4392-7

**Published:** 2019-09-11

**Authors:** Tormod Rimehaug, Karianne Framstad Holden, Stian Lydersen, Marit Sæbø Indredavik

**Affiliations:** 1 Regional Centre for Child and Youth Mental Health and Child Welfare, Department of Mental Health, Faculty of Medicine and Health Sciences, Norwegian university of science and technology (NTNU), Trondheim, Norway; 2 Child and adolescent psychiatry department, Nord-Trondelag Hospital Trust, Levanger, Norway; 3 Department of Clinical and Molecular Medicine, Faculty of Medicine and Health Sciences, Norwegian university of science and technology (NTNU), Trondheim, Norway

**Keywords:** Prevention, Substance, Mental health, Pregnancy outcome, Fetal growth restriction

## Abstract

**Backgrounds:**

In 2009, the prevention service “Familieambulatoriet” (FA) was established in three pilot hospital areas offering psychosocial support and health monitoring to parents in high risk regarding mental health and substance use, for the purpose of preventing child mental health and developmental problems through preschool years. This study selected new-born health as a preliminary endpoint for evaluation of population effects in three pilot areas, utilizing national statistics for birth cohorts from 2005 to 2013. *The aim* of the study is to evaluate changes in population new born health incidences associated with the establishment of new supportive and preventive FA-services at three pilot sites from 2009 in contrast to previous years and the remaining country. This quasi-experimental design evaluated changes in populations with new services available not those receiving the services, and controlled for national historical changes, variation between hospital districts, and random variation across the years before or years after the pilot services were introduced. Our hypothesis was to expect reduced frequencies of preterm births, SGA births, low APGAR scores, pediatric transfer, and new born abstinence symptoms in the pilot areas.

**Methods:**

The baseline was established through 4 years preceding 2009, contrasting changes at pilot sites the following 4 years 2009–2013 using the remaining hospital area populations in Norway 2005–2008 and 2009–2013 as contrasts.

**Results:**

Related to the introduction of FA services, we found three significant improvements in new born health using mixed effects logistic regression. 1) In the population rate of babies born prematurely with small for gestational age (SGA), using the 10th percentile criteria as the definition; odds ratio (OR) = 0.73 (95% Cl: 0.60 to 0.88). 2) A similar reduction using the 2.5th percentile criteria, although with wider confidence limits; OR = 0.73 (95% Cl: 0.54 to 0.99). 3) A decrease in the frequency of low APGAR scores (0–6) 5 min. after birth; OR = 0.80 (95% Cl: 0.68 to 0.95). Thus, the FA-areas remained significantly lowered on SGA rates or Low APGAR rates across the years after FA establishment, despite considerable variation, in contrast to the baseline years and to the remaining country. No significant effect was found for the outcomes frequency of premature births (unrelated to SGA), SGA among full-term babies, child abstinence symptoms or pediatric transfer of the baby. False negative findings may result from low-rate outcomes or studying the population rather than users.

**Conclusions:**

Population rates suggest that introducing FA services offering support and monitoring in high-risk families may contribute to improving aspects of new born infant health. Intervention components and strategies should be studied more closely using individual data.

**Electronic supplementary material:**

The online version of this article (10.1186/s12913-019-4392-7) contains supplementary material, which is available to authorized users.

## Background

The present study was conceived based on an unsystematic observation in a pediatric ward in 2015. “We see fewer premature babies in need for pediatric treatment now; is this a result of having Familieambulatoriet as part of our services?” To test this observation we wanted to evaluate changes in the incidence of preterm births, “small for gestational age” (SGA), APGAR scores, pediatric transfer of newborn babies and abstinence symptoms.

The scientific background of establishing “Familieambulatoriet” (FA) services was that it has been shown that previous and present maternal substance use and mental health problems represent risks related to a wide range of parental and child health issues, including fetal and subsequent child development. Several early selective preventive interventions have been developed in clinical practice to address these risks, including supportive follow-up services such as Familieambulatoriet in Copenhagen. In 2007, the Norwegian government challenged the health authorities to develop services that may prevent child developmental and mental health problems. One of the resulting initiatives was the pilot FA services at three sites named after their Danish inspiration, offering psychosocial support and health monitoring for child and parents in high-risk groups from pregnancy through birth, the post-natal period and preschool years. The FA pilots were established at three sites from 2009 and FA services were recommended for national implementation by the Norwegian Directorate of Health. This decision lacked a basis in effectiveness or outcome evaluation of the pilots. Only qualitative user evaluations, and evaluations of characteristics of the recruited families have been conducted [1].

The purpose of the FA services is to prevent and detect emerging child developmental and mental health problems from pregnancy through preschool years and to provide early intervention, based on support and monitoring. However, we have chosen to limit our study to intermediate outcomes related to pre- and postnatal health.

### Newborn health related to pregnancy and later development

Because of their known potential to indicate fetal and new born health as well as indicate risk for later unfavorable child development and mental health problems, incidence of preterm births, children born small-for-gestational-age (SGA), pediatric transfer, new born abstinence and APGAR scores were selected as outcomes. Besides, they are available in national statistics.

SGA is not defined by low birth weight in itself, but rather to be born small relative to gestational weeks, gender and parity. The combination of SGA and preterm birth indicates the possibility for pregnancy malnutrition, resulting in reduced fetal growth, immaturity of organs and possibly restricted brain development [2, 3]. Growth restrictions are further associated with later child motor, cognitive and language difficulties and delays, and with other developmental problems and mental health symptoms and disorders [4–9].

The typical “preterm behavioral phenotype” includes attention deficit hyperactivity disorder (ADHD) of the inattentive subtype, anxiety and autism spectrum traits with shyness and withdrawal, causing social difficulties [5, 10]. Preterm babies tend to show later problems with academic performance, employment, and general functioning. Their mental health problems may persist until adult age, which is also true for those born SGA at term [11–13]. Low birth weight is also significantly associated with increased new born, infant and in later life morbidity and mortality.

The APGAR score is affected by numerous fetal or maternal factors but also birth complications, including asphyxia, representing a risk for brain damage. The APGAR score can represent a global indicator of new born health [14], at 1 min. after birth more influenced by the birth process, and at 5 min. reflecting more of pregnancy factors and new born health. Abstinence symptoms in new born babies are severe symptoms signaling uterine exposure to addictive substances with potential neurodevelopmental consequences and immediate treatment needs [15, 16]. Direct transfer of the new born baby to pediatric care is the result of severe new born health problems requiring close observation or immediate care and may therefore be a global indicator of new born health.

SGA is associated to maternal lifestyle factors such as smoking and substance use, life stress or mental health problems, but also underlying disease conditions as infections and other medical conditions leading to inflammatory reactions in the fetus and placental dysfunction or [17–20]. Growth restrictions and preterm births are associated with maternal life stress, anxiety or depression [19, 20]. Maternal health has a less clear causal influence on later child mental health and development although its importance for fetal growth, premature birth, and SGA is known. These complex and interrelated links between parent and child, from conception through pre-school years represents a potential for improving future child health and development through improving maternal health and behavior by offering supportive health-promoting services and monitoring of care and development.

### The purpose of FAMILIEMBULATORIET

The original Familieambulatoriet (FA) was constructed as an out-reaching health-promoting and preventive intervention for pregnant women and their child selected by maternal substance use disorders, developed in Copenhagen by May Olofsson [21], inspired by the work of Dr. Loretta Finnegan at Thomas Jefferson University Hospital in Philadelphia, PA, USA. Their idea was to follow closely and support mothers with addictive disorders through their pregnancy and into the preschool years of their child to reduce medical and developmental risks for their children and to ensure early detection of child developmental or mental health problems eliciting secondary preventive and early intervention measures.

The Norwegian FA pilots expanded the recruitment to present and previous parental mental health and addictive risk. The interventions and strategies used are aiming to reduce stress and worry and increase confidence in the coming care challenges, enhancing self-care and support. This alliance building and support is based on maternal health evaluation during pregnancy, and later including relationship and child health and development evaluations. The procedures and strategies used to support and monitor the families are partly standardized but has not been tested as a package in RCT effectiveness studies. However many of the elements of support and monitoring of parent-child interaction and child development have been validated and tested for similar purposes.

### Aims, research strategy, and hypothesis

The aim of the study is to evaluate changes in new born health population incidences associated to the establishment of new supportive and preventive FA-services at three pilot sites.

The research strategy was to use mandatory collected data for births to the Medical Birth Registry of Norway (MBRN) representing the selected new born health outcomes to contrast sites and years with new FA-services in contrast to previous years and the remaining country to compensate for random and systematic variation across years and areas. Individual data or identification of actual users of FA-services were not available, but actual users were embedded in identified sub-populations. This allowed us to “expand” individual data for one variable in each analysis, using annual cohort grouping and grouping in areas (and the existence of new FA-services) as predictors.

Our hypothesis was to expect reduced frequencies of preterm births, SGA births, low APGAR scores, pediatric transfer, and new born abstinence symptoms.

## Methods

### Study strategy specified

We compared the incidence of selected health indicators in the population across five years 2009–2013 at FA-sites in contrast to baseline across four years 2005–2008 and the remaining country across nine years 2005–2013. This implies using newborn health as a preliminary endpoint rather than following child development through the preschool years. The study is not an effectiveness evaluation among those receiving the services, or of the specific effects of the invention components used in the FA services, but rather population changes in areas where any pregnancy with known risk indicators could be offered the additional services, which could be described as an “intention to select” sample.

This design controls the effect of introducing FA-services for random and systematic variation across years and areas, pooling data for three pilot sites (FA-sites) rather than evaluating each site separately. The minority of high-risk mothers actually receiving the services were therefore embedded in three identified sub-populations with FA-services.

### Data collection

Information was obtained from the Medical Birth Registry of Norway (MBRN) for the years 2005 to 2013. Aggregated data split for 26 hospital areas and nine years were delivered for the following variables: The frequency of premature births, low SGA scores, APGAR scores, pediatric transfer, and new born abstinence symptoms was requested from the Medical Birth Registry (MBRN). This registry contains standardized information about all births after 16 weeks of pregnancy at hospitals and birth clinics in the country, which encompasses more than 99.9% of all births. The MBRN files for the years 2005 to 2013 contain information from 545.156 registered births in Norway, with national birth cohorts of mean size 60.573. For each individual area included in this study, the Namsos, Levanger and Vestre Viken hospital areas, the annual birth cohorts varied from 402 to 10.556, reporting actual number for all variables, enabling us to calculate odds ratios and prevalence rates for any selection combining these levels; country (1), regions (5), counties (20), areas (26), separating six larger cities from their remaining county (district area), and the hospital areas with FA-services were separated from their remaining county. This sub grouped the data in 26 areas, of which three had FA sites the last five years established from late autumn 2008 to April 2009 – affecting birth cohorts from 2009. When pooling the population in the three FA-sites, 16.320 mothers had potential access to FA services across five years from 2009, on average 3446 mothers each year, and an estimated number of 816 unidentified mothers (5%) were recruited to FA services at three sites across five years.

The six cities, Oslo, Stavanger, Bergen, Trondheim, Bodø and Tromsø represented 32.1% of the national birth cohorts, and the 17 district areas without major cities represented 62.3% of the births. The three FA areas had a mix of urban areas, towns and rural areas (5.6% of the national birth cohorts) which were separated from their remaining county. The FA areas varied between city suburbs and small towns to rural areas, consisting of less than 50% urban areas, which is representative for the entire country.

The dominating majority of hospital services (especially pediatric and maternity wards) in Norway are organized by state-owned hospital trusts, dividing the counties into hospital areas where all citizens are zoned for a specific hospital, although patients can use other hospitals or be referred to specialized services at other hospitals. The majority of births take place at the local hospital (or during transport to a hospital), although 1.5% of all births take place at small local maternity homes, and approximately 150 give birth at home each year. The majority give birth at their local hospital, most expecting mothers visit their local family physician or a midwife for regular monitoring during the pregnancy, and virtually all births are registered in the MBRN connected to the mother’s homeplace.

### Service description

Pregnant women were offered FA services depending on their known addictive or mental health problems at the initiative of health personnel or by their own initiative based on a “low-threshold” principle. Those accepting enrollment received repeated outreach services for as long as the parents wanted to continue. The FA services continue as an outreach service offering repeated follow-up with support and monitoring towards school age. The mothers are invited to the FA services by health personnel in ordinary pregnancy services, based on known previous or present problems with mental health or substance use in their (extended) family. The recommended but voluntary follow-up services are available until the child enters school age, using follow-up contacts aimed at supporting the family and detecting concerns related to parents’ health, child development or care that may motivate evaluation, prevention or treatment.

The organizational structure around the services, personnel competence, and training, as well as the service components and procedures, do vary between sites. All units employed midwives, nurses, social workers and pedagogues as their primary personnel. The FA pilot units were interconnected with maternity and pediatric wards, gynecology, adult psychiatry, child psychiatry, and child rehabilitation teams at the local hospital, using office allocations, management responsibility, consultation, and personnel recruitment as strategies to ensure the “bridging” capability of the FA units.

The FA guidelines recommend the inclusion of both the mother and father/partner and focus on the needs for support for the risk factors for and challenges of parenthood and child development. FA services emphasize a supportive alliance and collaboration with the parents in the active monitoring of risk factors for and early signs of problems in care quality and development. Based on joint evaluation in dialogue, parents and children in need of extended services were offered contact with other services for assessment, prevention or treatment.

According to internal FA service statistics, the annual frequency of pregnant mothers offered FA services varied from 0.8 to 6.1% of the birth cohorts at one of the sites, and about half of these families stayed in contact with FA services for more than 6 months [1, 22].

### Variable definitions

The relevant variable definitions in the MBRN are as follows.

- Births are defined as pregnancies lasting more than 22 weeks or with a birthweight above 500 g.
- Full-term births are defined as occurring from the 37th week, earlier births are defined as preterm. Reduced birth weight is differentiated as low birth weight (LBW; birth weight < 2500 g), very low birth weight (VLBW; birth weight < 1500 g) or extremely low birth weight (ELBW; birth weight < 1000 g) SGA (small for gestational age) is defined by two criteria: A) Birth weight < 10th percentile relative to gestational age, gender, and parity, (SGA^10th^) or B) Birth weight < 2.5th percentile (SGA^2.5th^) [7]. The present study will distinguish between full-term and preterm born babies, using both the 10th and the 2.5th percentile as cut-off levels for SGA definition, since the SGA^10th^ group will include more genetically small children whereas the stricter criteria SGA^2.5th^ will exclude several children at significant risk.
- APGAR scores are structured evaluations of the state of the new born by a midwife or a physician one and five min. After birth (and later if necessary), following the APGAR system of scoring 0 to 2 on five observed items measuring appearance, pulse, grimace (response to stimulation), activity and respiration, summed to a score from 0 to 10 [23]. Scores of 0–6 are considered low scores, and scores of 0–3 indicate a critical state.
- Pediatric transfer is defined as the admission of the new born to a neonatal/pediatric unit directly from the maternity ward due to prematurity, respiratory problems, birth defects, perinatal infections or other somatic reasons.
- Abstinence describes the circumstance in which new born babies present with symptoms of abstinence from alcohol or prescribed or illegal drugs, as evaluated by physician/pediatrician.

### Statistics

We used mixed effects logistic regression analyses with one birth outcome at a time as the dependent variable, FA services and calendar year as fixed effect covariates, and area as a random effect. Years and historical trends are potential confounders. Birth outcomes were available but could only be analyzed at the individual level for one outcome in each analysis because the individual outcomes were generated by expanding aggregated data. The introduction of FA services was defined by the combination of area and the calendar years 2009 to 2013. Hence, the effect of FA services must be regarded as an “intention to select” effect, regardless of whether the mother followed the FA services or not.

All frequencies were calculated relative to the number of cases with available information, not the total number of births unless otherwise specified. The outcome variables had only 0.05 to 1% missing values.

Two-sided *p*-values < 0.05 were regarded as significant, and 95% confidence intervals are reported where relevant.

## Results

### Sample description

The three FA hospitals had 30.978 births over the nine years, which is 6.5% of the national total. The total birth rates per woman were from 1.65 to 2.00, with variations among years and areas, with lower birth rates in cities than in district areas.

The rate of married or cohabiting mothers was 92% throughout the period nationally, somewhat lower in district areas than in urban areas.

The variance of the random effect among the 26 areas was highly significant (*p* < 0.001) for all included health outcomes (see Table 1).

**Table 1:** Mixed model logistic regression with FA services from 2008 and calendar year (2005 to 2012) as covariates, and area as random effect

Dependent variable					
	Predictors	*OR*	*Log odds* ^*#*^	*95% CI*	*p*
Premature births	FA Service	.963		(.887 to 1.05)	.376
	Year	.983		(.979 to .987)	.001
	Area (random)		.004	(0.002 to 0.008)	.001
SGA10percentile Premature	**FA Service**	**.729**		**(.603 to .880)**	**.001**
	Year	.992		(.983 to 1.00)	.087
	Area (random)		.012	(.004 to .025)	.001
SGA2.5perctile Premature	**FA Service**	**.732**		**(.536 to .999)**	**.049**
	Year	.991		(.975 to 1.01)	.262
	Area (random)		.024	(.010 to .059)	.001
APGAR 1min. 0-6	FA Service	.948		(.857 to 1.05)	.301
	Year	.981		(.977 to .986)	.001
	Area (random)		.015	(.008 to .027)	.001
APGAR 5min. 0-6	**FA Service**	**.804**		**(.681 to .949)**	**.010**
	Year	1.01		(1.00 to 1.02)	.003
	Area (random)		.015	(.008 to .031)	.001
Abstinence	FA Service	1.22		(.723 to 2.05)	.461
	Year	.926		(.897 to .955)	.001
	Area (random)		.092	(.041 to .208)	.001
Pediatric transfer	FA Service	1.03		(947. to 1.12)	.478
	Year	.960		(.957 to .964)	.001
	Area (random)		.048	(.027 to .084)	.001

Note: ^#^ This is the variance (on a logarithmic odds scale) between the 26 areas for each outcome

Boldface types: Boldface types: Markes fixed effects of FA services reaching statistical significance (*p* < .05)

### Effect of FA services

Table 1 shows the results from mixed effect logistic regression analyses, with frequencies of new born health outcomes as dependent variables, predicted by years, areas (random factor) and FA services.

#### SGA

**Fig. 1:**
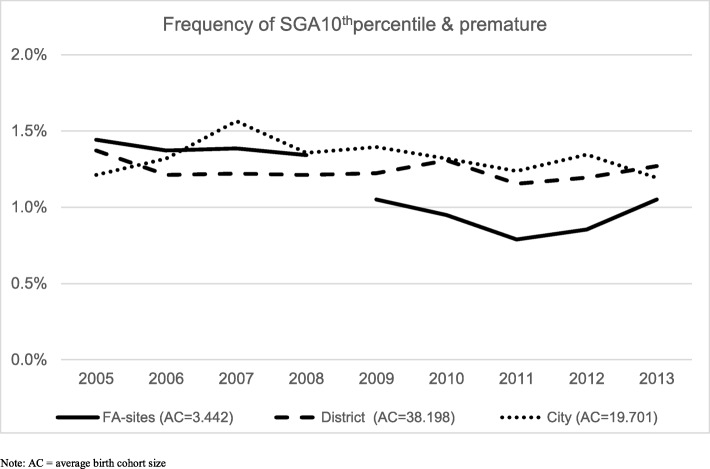
Annual frequency of SGA below 10^th^ percentile separately for the years 2005–2008 and 2009 to 2013, for the three FA sites combined, and other district or city hospital areas combined. Average birth-cohorts given in the legend. See Additional file 1: Table A1 for data details

**Fig. 2:**
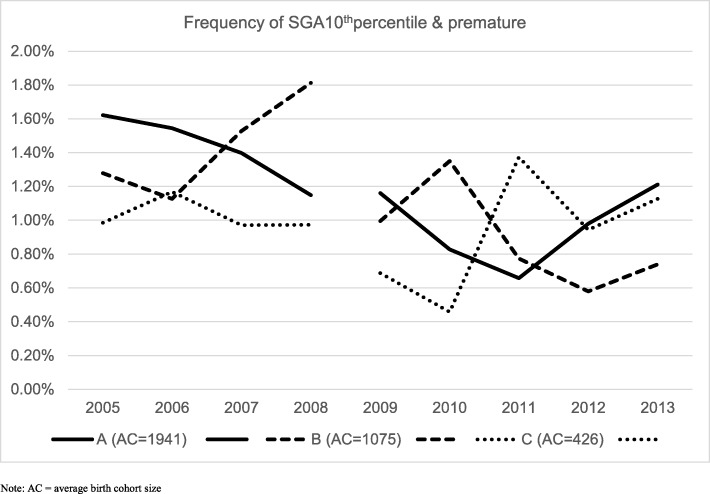
Annual frequency of SGA below 10^th^ percentile for the years 2005–2008 and 2009 to 2013, separately for three FA sites. Average birth cohort size given in legend. See Additional file 2: Table A2 for data details

The frequency of SGA10^th^p (premature and SGA babies) at FA sites decreased from an average of 1.30% in the years 2005 to 2008 to 0.92% at FA sites and 1.25% in other areas in 2009 to 2013. Figure 1 shows the development across years in the frequencies of premature birth & SGA for the FA sites and the remaining district or city areas in Norway, illustrating the mixed effect logistic regression for the population rate of babies born prematurely with SGA10^th^p; odds ratio (OR) = 0.73 (95% Cl: 0.60 to 0.88). No such effect was found for full-term babies. Figure 2 shows the annual SGA10^th^p split between the three FA sites to illustrate the variability across years ans sites. Data for these figures are specified in Additional file 1: Table A1 and Additional file 2: Table A2.

For SGA2.5^th^p among premature babies, the OR's for FA-sites were similar to those for the SGA10^th^p but with wider confidence limits, at the border of statistical significance although with wider confidence limits; OR = 0.73 (95% Cl: 0.54 to 0.99). No such effect was found for full-term babies.

#### Low APGAR score

The frequency of low APGAR 5 min. After birth was significantly reduced by FA services. The national average of low APGAR scores after 5 min. in the years 2005–2008 was 1.7% and remained at that level during the remaining years but dropped to 1.5% in the FA areas (see analysis details in Table 1). Th mixed effect logistic regression showed a significant effect on the frequency of low APGAR scores (0–6) 5 min. After birth; OR = 0.80 (95% Cl: 0.68 to 0.95), but not for APGAR scores at 1 min. After birth. APGAR scores were not separated for preterm and full-term births in MBRN, so we were not able to test any difference in effect for preterm and full-term.

All analyses of “FA-effects” were repeated using as contrasts only the remains of the two hospital regions with FA-sites. This improved the results somewhat, but not significantly, thus supporting the conclusions.

### Longitudinal population trends

Across the nine years included in the study, there was a population improvement trend in the frequency of premature babies (OR = .98), and reduced rate of new born abstinence symptoms (OR = .93). There was no significant general reduction in the rate of SGA among preterm or full-term babies, no significant general change in pediatric transfer and inconsistent longitudinal trends in the rate of low APGAR-scores.

## Discussion

Establishing FA services at three pilot sites in 2009 was associated with significant local population improvements in new born baby health, as measured by annual SGA rates among premature babies (10th and 2.5th percentile cut-offs) and the frequency of low APGAR scores 5 min. after birth. We found FA-effects with ORs in the range of 0.7 to 0.8 on these health outcomes following the initiation of new services, pooling four years before and five years after the FA introduction. No significant FA effect was found for SGA among full-term babies, the rate of preterm births, child abstinence or pediatric transfer. These results are population changes in the areas where FA services were introduced, not individual changes among those actually receiving the services, which might be termed as an "intention to select" effect.

It is possible that the reductions in SGA and low APGAR scores related to the establishment of FA-services can be explained by influences on health-related behaviors, stress, and confidence among mothers receiving the services. Similar manualized supportive services, such as the Nurse-Family Partnership (NFP), have been shown to influence mother/baby health at birth, later child development, parental education completion, work participation, parenting, partner violence, and health-related behaviors [24]. However, SGA and APGAR scores have not been specifically studied as outcomes of NFP. Finding no influence on the rate of preterm birth is not in accordance with an observational study of FA users at one FA site [1] and studies on the NFP [24], also showing higher levels of preterm births in the target group but a decrease among those offered supportive services. This could indicate that the preterm rates in the population are more associated with factors not associated with the FA target groups. Studying those receiving services could give different results than when studying the population.

An issue regarding statistical sensitivity/power is illustrated by the difference between SGA using the 10^th^ or 2.5^th^ percentile as criteria. The lower rates using the 2.5^th^ percentile criteria resulted in wider CI ranges and a barely significant effect, although the OR is similar to that for the 10^th^ percentile (both ORs average at − 0.73).

Finding a FA effect on 5 min. APGAR scores but not 1 min. APGAR scores is an expected result. The 1 min. score will be largely influenced by the birth process affecting the baby’s immediate state, whereas the 5 min. score tend to reflect more factors that have been present before the birth and do not decline after birth. Therefore, a low 5 min. score may indicate fetal risk factors, possibly related to maternal lifestyle.

The variations between the FA sites and between years with FA-services shown in Fig. 2 illustrate the large variations, and the importance of pooling several years and several sites when evaluating these outcomes. A possible trend toward reduced FA-effect at the end of the study period should motivate a more thorough study of whether the effects are retained in later years, examining the mediators and moderators of importance for achieving an maintaining the effects that FA services seem to have the potential to create on maternal and fetal health across pregnancy and birth.

### Strengths and limitations

The strongest aspect of this study is the large database MBRN that contains only 0.1% missing data, are collected routinely, and are considered to give high-quality new born health information at the population level. The selected years are not contaminated by the introduction of other similar services or interventions to FA since the decision to implement similar services came at the end of the study peiod in 2013, and is still not effective nationally. However, some areas did have services similar to FA in activity across the entire study period. The pooling of hospital areas and years compensated for random or systematic variability across areas and years illustrated in Fig. 2.

The related limitation is that the aggregated data limited the possibility of investigating covariation across outcomes and studying complex prediction models with moderators and mediators. However, none of the available variables showed a significant association to FA services indicating a possible confounding effect at aggregated level.

A major limitation is that we have studied aggregated data, limiting the possibility to analyze individual associations, moderators and confounders. Another limitation of the study is that the outcome variables are dichotomous, for some reflecting relatively low-frequency events that require large samples to produce statistically reliable and significant results. This is illustrated by the difference between the SGA10^th^ and SGA2.5^th^ criteria regarding a FA effect, showing differences only in statistical probability but not in effect size. The lack of a manualized intervention content makes it difficult to draw conclusions about which factors were effective in eliciting the effects associated with FA services. The study does not evaluate the effectiveness of the intervention components or actual user outcomes.

Systematic descriptions of how services should be conducted would also be vital to monitor and improve the quality of services. Finally, too little information was available regarding the quality of ordinary services in Norway. These services may have increased the variability and reduced some contrast between areas but did not affect the pre-post control element in our design.

The results should be interpreted cautiously, as the study did not specifically study mothers actually using the FA services, only population incidences where such services were introduced. However, such population effects are the intended political outcome of investing in FA services. The study should be followed up using individual data from the MBRN or actual users of FA services and investigating associations between outcomes and multiple risk factors. SGA reduction and improved APGAR could be results of similar underlying improvements in maternal health. Furthermore, studies should follow parents and children further, until school age to fully assess outcomes for families with and without access to preventive services. Finally, a new study should be conducted to see if the effects at the population level are retained through several years of FA services, including also FA-sites established after 2013.

Studying population changes while actual services reached only a small percentage of the population will risk false negative conclusions regarding some effects of the FA services. As an example, a clinical study at one FA site showed almost the double frequency of premature births among FA users compared to the local population [1].

### Clinical implications

There is a potential for improving new born health through preventive interventions aimed at supporting a healthy lifestyle and preparing for parenthood, targeting pregnant women at risk due to mental health or substance use problems. This effort may represent a first step in the prevention of later child developmental and mental health problems. However, such preventive efforts should use manualized procedures evaluated for their effectiveness to enable quality assurance.

## Conclusion

Population incidence suggests that supportive interventions, such as the FA services during pregnancy targeting high-risk groups regarding mental health and substance problems, may reduce the frequency of SGA among preterm babies and low APGAR (5 min.) scores among new born infants. The pilot services investigated in the present study did not result in significantly fewer babies born preterm or with abstinence symptoms. Some results may be falsely negative due to low baseline rates or because this study evaluated the entire population, not only the users of FA services. Intervention components and strategies in FA should be systematized and studied more closely using individual data including confounders and mediators in the analyses. The effects on SGA and APGAR scores should be replicated for FA services established after 2013 and evaluated at pilot sites for stability in the years after 2013.

## Additional files

Additional file 1: **Table A1.** Data for Fig. 1. Separately for each year and separated for FA sites, district areas and cities summed; number of premature births with SGA10^th^ percentile, actual number in birth cohort and rate of SGA10^th^. (DOCX 17 kb)

Additional file 2: **Table A2.** Data for Fig. 2. Separately for each year and each FA site; number of premature births with SGA10^th^ percentile, actual number in birth cohort and rate of SGA10^th^. (DOCX 17 kb)

## Data Availability

The data that support the findings of this study are available from [third party name] but restrictions apply to the availability of these data, which were used under license for the current study, and so are not publicly available. Data are however available from the authors upon reasonable request and with permission of the Medical Birth Registry of Norway.

## Abbreviations

CAMHS: Child and adolescent mental health services at hospital
FA: Familieambulatoriet
MBRN: Medical Birth Registry of Norway
SGA: Small for Gestational Age
