# Supplementary material for: “Five-year changes in population newborn health associated with new preventive services in targeted risk-group pregnancies”
Source: BMC Health Serv Res. 2019 Sep 11;19:658. doi: 10.1186/s12913-019-4392-7 (PMC6737636; doi:10.1186/s12913-019-4392-7)
Supplement: Supplementary file 1 — Table A1. Data for Fig. 1. Separately for each year and separated for FA sites, district areas and cities summed; number of premature births with SGA10th percentile, actual number in birth cohort and rate of SGA10th. (DOCX 17 kb) [file 12913_2019_4392_MOESM1_ESM.docx]

**ADDITIONAL TABLE A1**

Table A1 - Data for Figure 1.

Separately for each year and separated for FA sites, district areas and cities summed; number of premature births with SGA10^th^ percentile, actual number in birth cohort and rate of SGA10^th^.

|  |  | 2005 | 2006 | 2007 | 2008 | 2009 | 2010 | 2011 | 2012 | 2013 | Annual  average |
| --- | --- | --- | --- | --- | --- | --- | --- | --- | --- | --- | --- |
| FA site areas | SGA10^th^ & premature | 51 | 48 | 47 | 46 | 37 | 33 | 26 | 29 | 36 | 39 |
| (*AC*=3.442) | Birth cohorts | 3535 | 3500 | 3391 | 3430 | 3522 | 3481 | 3296 | 3398 | 3425 | 3442 |
|  | Percentages | 1,4% | 1,4% | 1,4% | 1,3% | 1,1% | 0,9% | 0,8% | 0,9% | 1,1% | 1,14% |
| District areas | SGA10^th^ & premature | 498 | 452 | 457 | 469 | 486 | 514 | 445 | 462 | 478 | 473 |
| (small towns & rural) | Birth cohorts | 36315 | 37311 | 37459 | 38694 | 39733 | 39330 | 38566 | 38709 | 37662 | 38198 |
| (*AC*=38.198) | Percentages | 1,4% | 1,2% | 1,2% | 1,2% | 1,2% | 1,3% | 1,2% | 1,2% | 1,3% | 1,22% |
| City areas | SGA10^th^ & premature | 222 | 252 | 299 | 272 | 286 | 271 | 249 | 269 | 233 | 261 |
| (*AC*=19.701) | Birth cohorts | 18320 | 19109 | 19108 | 20046 | 20514 | 20542 | 20126 | 20000 | 19543 | 19701 |
|  | Percentages | 1,2% | 1,3% | 1,6% | 1,4% | 1,4% | 1,3% | 1,2% | 1,3% | 1,2% | 1,30% |
| Note: AC = average birth cohort size |  |  |  |  |  |  |  |  |  |  |  |
