# Supplementary material for: “Five-year changes in population newborn health associated with new preventive services in targeted risk-group pregnancies”
Source: BMC Health Serv Res. 2019 Sep 11;19:658. doi: 10.1186/s12913-019-4392-7 (PMC6737636; doi:10.1186/s12913-019-4392-7)
Supplement: Supplementary file 2 — Table A2. Data for Fig. 2. Separately for each year and each FA site; number of premature births with SGA10th percentile, actual number in birth cohort and rate of SGA10th. (DOCX 17 kb) [file 12913_2019_4392_MOESM2_ESM.docx]

**ADDITIONAL TABLE A2**

Table A2 - Data for Figure 2. Separately for each year and FA site; number of premature births with SGA10^th^ percentile, actual number in birth cohort and rate of SGA10^th^.

|  |  | 2005 | 2006 | 2007 | 2008 | 2009 | 2010 | 2011 | 2012 | 2013 | Annual  average |
| --- | --- | --- | --- | --- | --- | --- | --- | --- | --- | --- | --- |
| FA site A (AC=1941) | SGA10^th^ & premature | 33 | 31 | 27 | 22 | 23 | 16 | 12 | 19 | 23 | 23 |
|  | Birth cohorts | 2034 | 2007 | 1932 | 1916 | 1981 | 1933 | 1825 | 1938 | 1899 | 1941 |
|  | Percentages | 1,62 % | 1,54 % | 1,40 % | 1,15 % | 1,16 % | 0,83 % | 0,66 % | 0,98 % | 1,21 % | 1.17 % |
| FA site B (AC=1075) | SGA10^th^ & premature | 14 | 12 | 16 | 20 | 11 | 15 | 8 | 6 | 8 | 12 |
|  | Birth cohort | 1095 | 1065 | 1047 | 1103 | 1105 | 1111 | 1034 | 1036 | 1082 | 1075 |
|  | Percentages | 1,28 % | 1,13 % | 1,53 % | 1,81 % | 1,00 % | 1,35 % | 0,77 % | 0,58 % | 0,74 % | 1,13 % |
| FA Site C (AC=426) | SGA10^th^ & premature | 4 | 5 | 4 | 4 | 3 | 2 | 6 | 4 | 5 | 4 |
|  | Birth cohorts | 406 | 428 | 412 | 411 | 436 | 437 | 437 | 424 | 444 | 426 |
|  | Percentages | 0,99 % | 1,17 % | 0,97 % | 0,97 % | 0,69 % | 0,46 % | 1,37 % | 0,94 % | 1,13 % | 0,97 % |
| Note: AC = average birth cohort size | |  |  |  |  |  |  |  |  |  |  |
